# Supplementary material for: Memory consolidation and improvement by synaptic tagging and capture in recurrent neural networks
Source: Commun Biol. 2021 Mar 3;4:275. doi: 10.1038/s42003-021-01778-y (PMC7977149; doi:10.1038/s42003-021-01778-y)
Supplement: Supplementary file 1 — Supplementary Information [file 42003_2021_1778_MOESM1_ESM.pdf]

Supplementary Information:

Memory consolidation and improvement by synaptic tagging and capture in recurrent neural networks

Jannik Luboeinski [1,2,*], Christian Tetzlaff [1,2,#]

[1] Department of Computational Neuroscience, III. Institute of Physics – Biophysics, University of Göttingen, Göttingen, Germany

[2] Bernstein Center for Computational Neuroscience, Göttingen, Germany

Correspondence:

* jannik.luboeinski@phys.uni-goettingen.de

# tetzlaff@phys.uni-goettingen.de

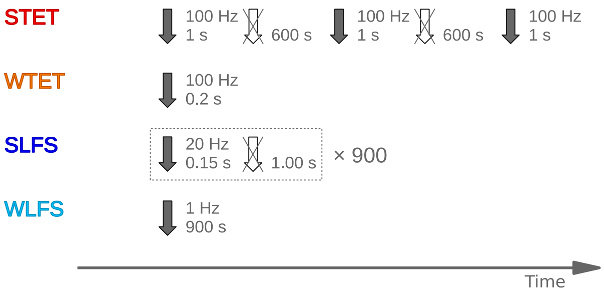

Supplementary Figure S1: Standard protocols for the the induction of early- and late-phase synaptic potentiation and depression as used in [1]. Filled arrows indicate stimulation at the specified frequency for the specified time, crossed arrows indicate breaks for the specified time.

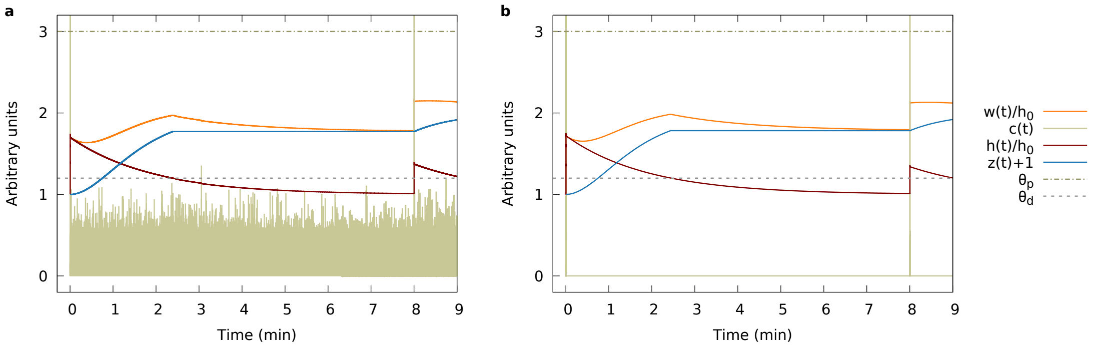

Supplementary Figure S2: Matching temporal dynamics of the weight of a synapse in **(a)** a spiking network simulation run in full detail and **(b)** an accelerated network simulation with spiking dynamics only during relevant learning- and retrieval-related periods. Outside of these periods, the fully-simulated calcium concentration stays below the plasticity thresholds and thus, can be neglected. Parameter setting: $w_{\text{ie}}/h_0 = 4$, $w_{\text{ii}}/h_0 = 4$, $n_{\text{CA}} = 150$.

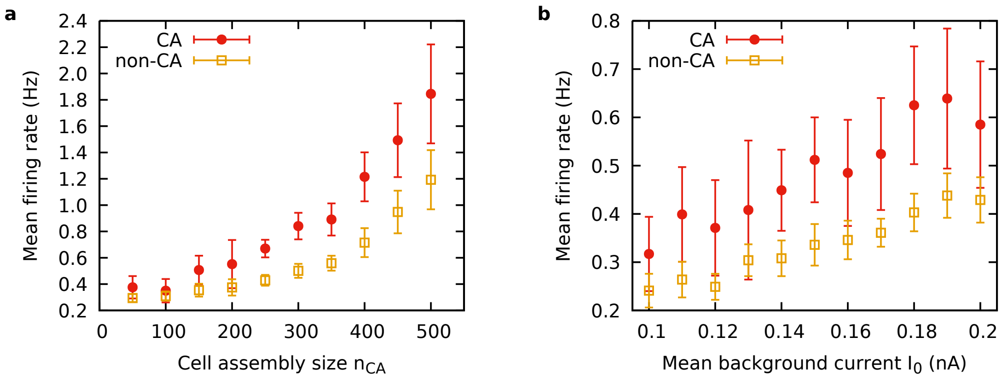

Supplementary Figure S3: **(a)** The mean standby firing rate in the excitatory population of a network holding one cell assembly grows strictly monotonically with the size of the assembly (measurement 10 seconds after learning the assembly). Firing rates within the assembly (CA) are higher than in the rest of the population (non-CA). The firing rates for small- to medium-sized assemblies are around the average physiological value of 0.5–1.0 Hz for pyramidal cells in the hippocampus during exploratory wake states [2, 3]. Mean background current: $I_0 = 0.15\,\mathrm{nA}$. **(b)** Variation of the mean background current causes the mean firing rate to rise slightly. Assembly size: $n_{\mathrm{CA}} = 150$. Error bars indicate the standard deviation across ten trials. Other parameters: $w_{\mathrm{ie}}/h_0 = 4$, $w_{\mathrm{ii}}/h_0 = 4$.

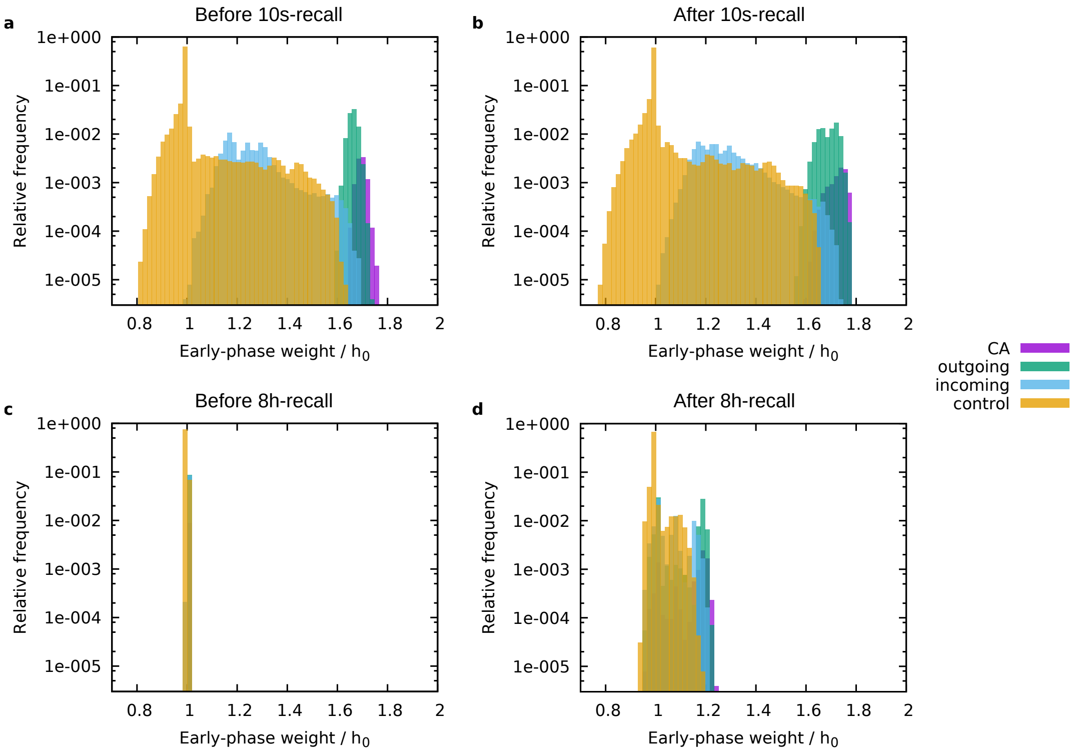

Supplementary Figure S4: Distribution of early-phase weights in different groups of synapses of one sample network. **(a,b)** Ten seconds after learning, the weights within the cell assembly ('CA') are all increased significantly, as well as the weights outgoing from assembly neurons ('outgoing'). The weights within the control population ('control') stay mostly low, while there is a larger fraction of weights incoming to assembly neurons from control neurons ('incoming') that are increased. **(c,d)** After eight hours, the initially learned structure is not anymore reflected by the early-phase weights (cf. Fig. S5 for the late-phase weights). Moreover, the early-phase weights are only slightly affected by 10s-recall (compare **(a)** and **(b)**). By contrast, 8h-recall causes a significant change in the distribution of early-phase weights (compare **(c)** and **(d)**). In each plot, the weights were discretized into 100 bins and the relative frequencies were normalized across all groups of synapses. Note that in the given units, the initial value of early-phase weights is unity. Parameters: $n_{\mathrm{CA}} = 150$, $w_{\mathrm{ie}}/h_0 = 4$, $w_{\mathrm{ii}}/h_0 = 4$.

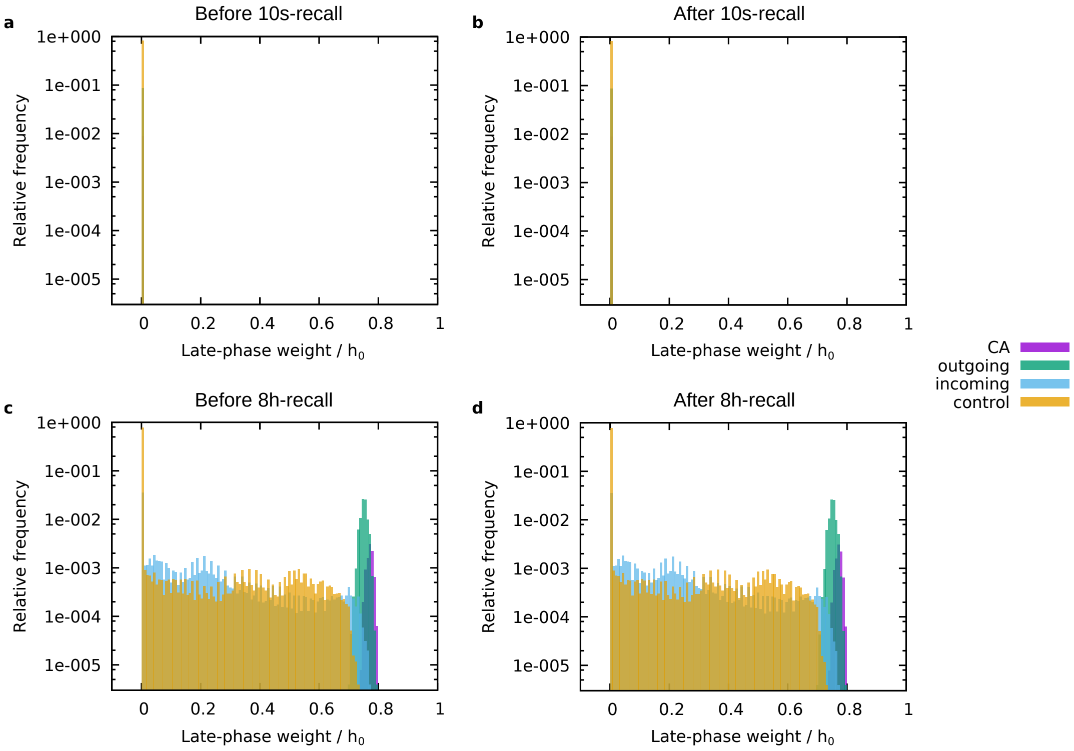

Supplementary Figure S5: Distribution of late-phase weights in different groups of synapses of one sample network. **(a,b)** Due to their long timescales, the late-phase weights are not directly affected by learning or recall (cf. Fig. S4). Hence, there is also no substantial change during 10s-recall (compare **(a)** and **(b)**) or during 8h-recall (compare **(c)** and **(d)**). **(c,d)** Across eight hours, however, late-phase weights are significantly elevated and reflect the consolidated cell assembly. In each plot, the weights were discretized into 100 bins and the relative frequencies were normalized across all groups of synapses. Note that the initial value of late-phase weights is zero. Parameters: $n_{\mathrm{CA}} = 150$, $w_{\mathrm{ie}}/h_0 = 4$, $w_{\mathrm{ii}}/h_0 = 4$.

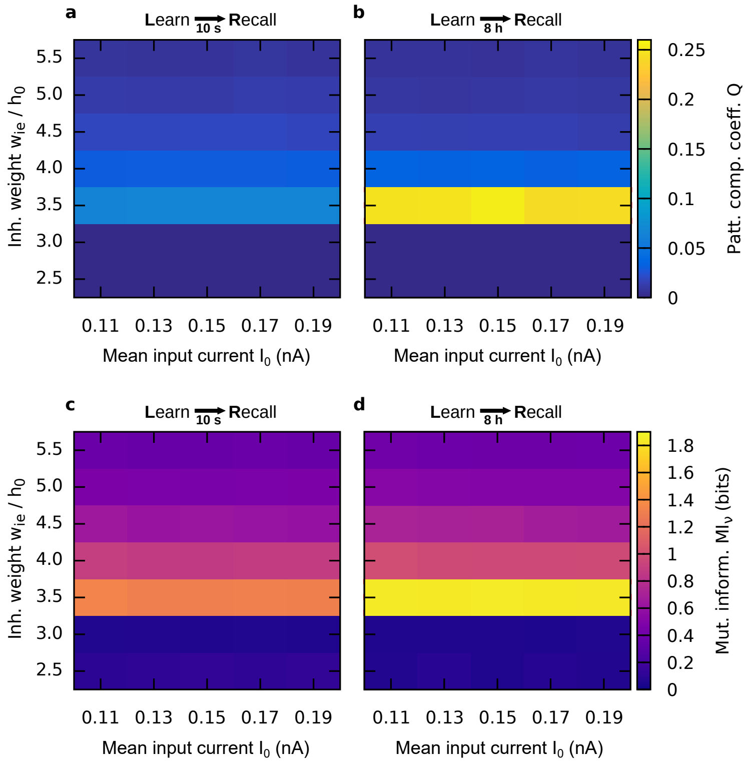

Supplementary Figure S6: Quality of the recall 10 s and 8 h after learning as a function of the I→E (w_{ie}) coupling strength and the mean background current I_0. **(a,b)** Pattern completion coefficient Q (with non-significant values set to zero, see Methods); **(c,d)** mutual information MI_ν between the neuronal activities in the network during learning and during recall. Values were averaged across ten trials. Parameters: $n_{\mathrm{CA}} = 150$, $w_{\mathrm{ii}}/h_0 = 4$.

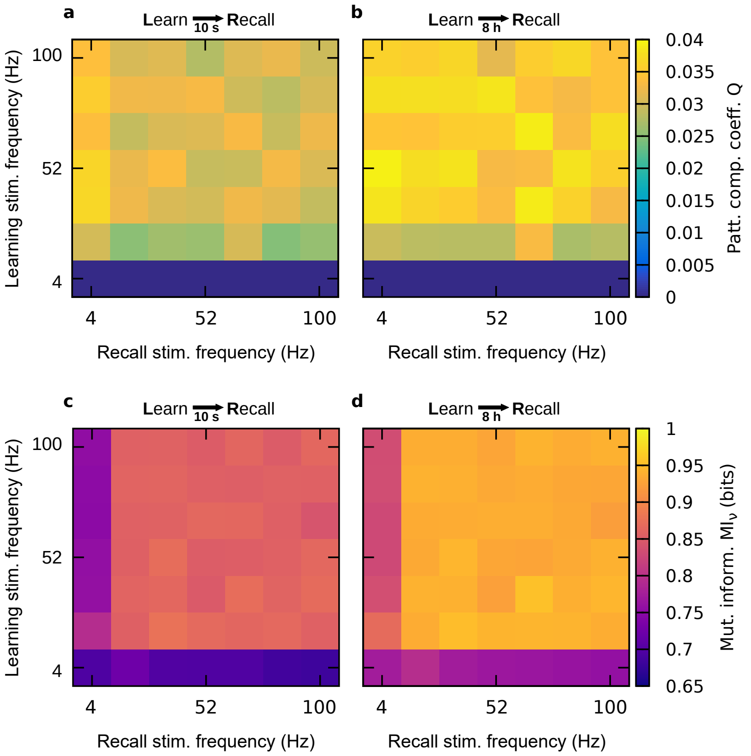

Supplementary Figure S7: Quality of the recall 10 s and 8 h after learning as a function of the stimulation frequency f_{stim}, which is varied separately for learning and recall. **(a,b)** Pattern completion coefficient Q (with non-significant values set to zero, see Methods); **(c,d)** mutual information MI_{ν} between the neuronal activities in the network during learning and during recall. Values were averaged across 100 trials. Parameters: $n_{\mathrm{CA}} = 150$, $w_{\mathrm{ie}}/h_0 = 4$, $w_{\mathrm{ii}}/h_0 = 4$.

Supplementary References

[1] Li, Y., Kulvicius, T. & Tetzlaff, C. Induction and consolidation of calcium-based homo- and heterosynaptic potentiation and depression. *PLOS One* **11**, e0161679 (2016).

[2] Mizuseki, K. & Buzsáki, G. Preconfigured, skewed distribution of firing rates in the hippocampus and entorhinal cortex. *Cell Rep.* **4**, 1010–1021 (2013).

[3] Mizuseki, K. & Miyawaki, H. Hippocampal information processing across sleep/wake cycles. *Neurosci. Res.* **118**, 30–47 (2017).
