# Supplementary material for: Memory consolidation and improvement by synaptic tagging and capture in recurrent neural networks
Source: Commun Biol. 2021 Mar 3;4:275. doi: 10.1038/s42003-021-01778-y (PMC7977149; doi:10.1038/s42003-021-01778-y)
Supplement: Supplementary file 2 — Description of Supplementary Files [file 42003_2021_1778_MOESM2_ESM.pdf]

Description of Additional Supplementary Files

File name: Supplementary Data 1

Description: Source data for Figure 4, Figure 5, Figure 7, and Figure 9.

File name: Supplementary Data 2

Description: Source data for Figure 2, Figure 3, and Figure 6.
